# Comparing hydrazine-derived reactive groups as inhibitors of quinone-dependent amine oxidases

**DOI:** 10.1080/14756366.2016.1265518

**Published:** 2017-01-23

**Authors:** Ashley A. Burke, Elizabeth S. Severson, Shreya Mool, Maria J. Solares Bucaro, Frederick T. Greenaway, Charles E. Jakobsche

**Affiliations:** Carlson School of Chemistry and Biochemistry, Clark University, Worcester, MA, USA

**Keywords:** Lysyl oxidase, hydrazine, enzyme kinetics, irreversible inhibition

## Abstract

Lysyl oxidase has emerged as an important enzyme in cancer metastasis. Its activity has been reported to become upregulated in several types of cancer, and blocking its activity has been shown to limit the metastatic potential of various cancers. The small-molecules phenylhydrazine and β-aminopropionitrile are known to inhibit lysyl oxidase; however, issues of stability, toxicity, and poorly defined mechanisms limit their potential use in medical applications. The experiments presented herein evaluate three other families of hydrazine-derived compounds – hydrazides, alkyl hydrazines, and semicarbazides – as irreversible inhibitors of lysyl oxidase including determining the kinetic parameters and comparing the inhibition selectivities for lysyl oxidase against the topaquinone-containing diamine oxidase from lentil seedlings. The results suggest that the hydrazide group may be a useful core functionality that can be developed into potent and selective inhibitors of lysyl oxidase and eventually find application in cancer metastasis research.

## Introduction

Lysyl oxidase (LOX) is an enzyme whose activity is associated with multiple diseases including metastatic cancer, stiffening of connective tissue^1^, myocardial fibrosis^1^, chronic liver disease^2^, and hepatic fibrosis^3^. The correlation between high LOX activity and cancer metastasis is strong enough that upregulated LOX activity can be used as a diagnostic marker for the severity of cancer in patients. For example, higher LOX levels in human lung adenocarcinomas correlate with higher tumor invasiveness and lower 5-year patient survival rates^4^. Similarly, in human head and neck squamous cell carcinomas, higher levels of LOX correlate with later stage cancers, increased metastasis to the lymph node, and decreased overall patient survival^5^. In addition to LOX, there are four LOX-like enzymes (LOXL1–LOXL4) whose catalytic domains are highly conserved, but whose N-terminal domains enable unique biological functions^6^. LOXL2 also shows increased expression in a variety of cancers^7^.

For many types of cancer, increased LOX activity promotes metastasis of cancer cells and progression of tumor growth. For example, El-Haibi et al. have shown that stimulating breast cancer cells to increase LOX synthesis activates those cells into a metastatic phenotype^8^. Cox et al. have shown that metastasis of cancer cells can be promoted by the secretion of enzymatically active LOX by neighboring fibroblast cells^9^. This effect is attributed to LOX's normal catalytic function, which promotes the crosslinking of collagen, modifies the extracellular matrix, and creates a microenvironment that is favorable for cell migration. Baker et al. have shown that treating colorectal cancer cells with LOX increases their propensity to metastasize and also to undergo angiogenesis after forming solid tumors^10^. The authors attribute these effects to upregulation of the angiogenesis-promoting VEGF pathway. Additional results indicate that LOX can increase cellular metastasis by activating focal adhesion kinase and Src kinase pathways and by increasing hydrogen peroxide levels^11^. By contrast, some gastric cancers suppress LOX activity^12^, which suggests that LOX's biology is quite complex.

Blocking LOX's enzymatic function can reduce migration of cancer cells. Using siRNA to knock down LOX expression can significantly reduce the hypoxia-induced invasiveness of nonsmall-cell lung cancer cells^13^. Inhibiting LOX's catalytic activity in cervical cancer cells can reduce their invasion and migration and also block the epithelial–mesenchymal transition, which is associated with metastatic phenotypes^14^. Additionally, inhibiting LOX can decrease metastasis of human breast cancer-derived tumors in mice^15^.

**Figure 1.**
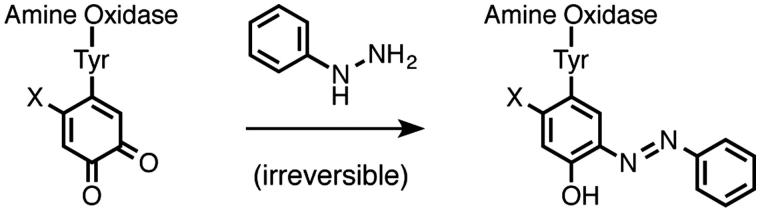
Overview of quinone-containing amine oxidase cofactors and inhibition by phenyl hydrazine. X = OH for topaquinone; X = lysine-ɛNH for lysyl tyrosylquinone.

LOX performs its enzymatic function, converting primary amines into aldehydes, by using a quinone cofactor and a redox-active copper ion. Specifically, the quinone cofactor is lysyl tyrosylquinone (LTQ) (Figure 1), which is structurally distinct from the topaquinone cofactor found in most other copper-containing amine oxidases. Our previous contributions to understanding the structural biology of LOX include identifying the histidine residues that serve as copper-binding ligands^16^ and identifying the cysteine pairs that form disulfide bridges to stabilize LOX's structure^17^. Although several crystal structures have been reported for various topaquinone-containing amine oxidases, some with covalently-bound hydrazine inhibitors^18^, very little is known about the structure of LOX and the nature of its active site. It has been hypothesized, however, that since LOX's natural substrates are proteins, the active site must be open and more like that of the *Pichia pastoris* amine oxidase^19^ rather than a narrow substrate channel like those typically found in topaquinone-containing amine oxidases.

**Figure 2.**
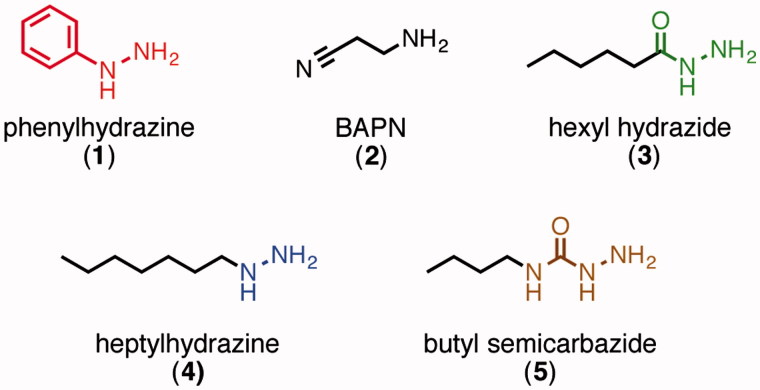
Inhibitor structures.

It has been well established that small-molecule aryl hydrazines, especially phenylhydrazine^1^, can act as irreversible inhibitors for LOX and most other quinone-containing amine oxidases^20–23^. Indeed, LOX's LTQ cofactor was originally identified by mass spectrometry and Edman degradation analysis of the adduct formed between LOX and ^14^C-phenylhydrazine^24^. The kinetics of inhibition of LOX by phenylhydrazine have been previously studied by Williamson et al., and these data show that the inhibition is competitive with substrate and irreversible^25^. Studies of a synthetic small-molecule model of LTQ have shown that the phenylhydrazine adduct preferentially tautomerizes into its azo form (Figure 1)^26^. Phenylhydrazine, however, is known to have low target specificity and to readily decompose under biological conditions, which limits its potential use for *in vivo* applications. Other types of hydrazine-containing organic molecules that are more stable than phenylhydrazine, such as alkyl hydrazines, hydrazides, and semicarbazides (Figure 2), have not been studied much as LOX inhibitors. Some of these structures have, however, been investigated as inhibitors of other copper-containing amine oxidases.

The most established LOX inhibitor is β-aminopropionitrile (BAPN, **2**, Figure 2). Although this molecule does covalently attach to the enzyme in a time-dependent manner, its kinetics do not follow those expected for direct irreversible inhibition^27^^,^^28^. Indeed, its mechanism of action, which is not yet fully understood, is thought to be by irreversible modification of an active-site residue by the aldehyde product^28^. BAPN treatment has also been reported to have some side effects, and it is not approved for clinical use^29^.

We set out to evaluate the effectiveness and specificity of various organic hydrazine derivatives to help provide a basis for developing new selective irreversible inhibitors of LOX. These studies directly compare four families of organic hydrazine compounds as inhibitors of LOX and also of lentil seedling diamine oxidase (LSDAO), which was chosen as a representative topaquinone-containing amine oxidase with a somewhat restrictive substrate-binding channel for comparison. Representative members of the alkyl hydrazine, hydrazide, and semicarbazide families (**3–5**) were synthesized and compared to phenylhydrazine, including analyses of their kinetic parameters for irreversible inhibition. These comparisons of inhibition parameters and selectivity provide a basis for deciding which of these “core” inhibitor structures are best suited as starting points for development of more complex molecules that could act as potent and selective inhibitors of LOX and also have favorable pharmacokinetic properties. Such inhibitors would be important not only as potential drug candidates, but also as chemical tools to study LOX-related biological mechanisms that are involved in cancer metathesis and other diseases.

## Materials and methods

### LSDAO extraction

The LSDAO extraction process was carried out at 4 °C according to the method of Floris et al.^30^ with slight modifications. Eight-day-old lentil seedlings (*Lens esculenta*) were blended and extracted with phosphate buffer (5 mM, pH 6.0, buffer A). Ammonium sulfate (230 g/L, 40% saturation) was used to precipitate contaminants, and then additional ammonium sulfate (125 g/L, 60% saturation) was used to precipitate the LSDAO. The crude precipitate was redissolved, dialyzed against buffer A, applied to a DEAE column, and eluted with buffer A. Active fractions were adjusted to pH 6.5, applied to a CM resin column, and eluted with 5 mM phosphate buffer, pH 6.5. Active fractions were concentrated and the buffer was changed to 10 mM phosphate, pH 7.0 before the protein was applied to a Sephacryl S-300 column and eluted with the same buffer. The concentration of the LSDAO dimer was determined from its absorption at 278 nm in 0.1 M phosphate buffer^31^ using a molar extinction coefficient of 2.45 × 10^5^ M^−1 ^cm^−1^. The enzyme had very high activity (more than 1 μkat/mg protein). Analysis by a 7.5% SDS PAGE gel showed a single band.

### LOX extraction

LOX was isolated from bovine aorta at 4 °C using slight modifications of previously published procedures^32^. All steps were carried out at in 16 mM phosphate, pH 7.8 (buffer B). After blending the aorta in buffer B containing 400 mM NaCl and 1 mM EDTA to remove soluble contaminants, the enzyme was extracted with buffer B containing 4 M urea and 1 mM EDTA. The crude enzyme extract was filtered, diluted with an equal volume of buffer B to obtain a urea concentration of 2 M, and loaded onto a DEAE column. After washing with buffer B containing 400 mM NaCl, the enzyme was eluted with buffer B containing 400 mM NaCl and 6 M urea. The urea concentration was reduced to 4 M by adding buffer B, and the solution was added to a slurry of ceramic hydroxyapatite in buffer B containing 4 M urea. After stirring, the supernatant was removed by filtration and dialyzed overnight against buffer B. The solution was added to a slurry of ceramic hydroxyapatite in buffer B, washed with small portions of 1 M phosphate and then buffer B, and then eluted with buffer B containing 6 M urea and 500 mM NaCl. After concentration to about 20 mL, the protein was applied to a Sephacryl S-200 gel filtration column preequilibrated with buffer B containing 50 mM NaCl, 1 mM EDTA, and 6 M urea and eluted with the same buffer. Active fractions were concentrated to 1 mL. Total protein concentration for 6 M urea solutions^33^ was determined by its absorption at 280 nm using a molar extinction coefficient of 8.39 × 10^4^ M^−1 ^cm ^−1^. SDS-PAGE and mass spectrometric analysis indicated that the isolated LOX was mostly pure, but also included a small amount (∼5%) of a 22 kDa protein impurity that has been identified as tyrosine-rich acidic matrix protein, TRAMP, which is unreactive under the activity assay conditions^34^. The enzyme was stored as a concentrated solution at 4 °C and was used immediately as it loses its activity very rapidly (complete loss within 10 days).

### Activity assay

The activity assay is adopted from the method described by Palamakumbura et al.^35^. Enzyme and inhibitor were incubated at 37 °C for the desired inhibition time, diluted into the assay mixture, and analyzed by fluorescence (Agilent Technologies Cary Eclipse Fluorescence Spectrometer, Cary Eclipse Software, 37 °C, λ_ex_ = 568 and λ_em_ = 581 nm). The assay was carried out in boric acid buffer (50 mM, pH 8.2) with 1.2 M urea for LOX or in phosphate buffer (0.1 M, pH = 7.0) for LSDAO. The final reagent concentrations in the assay mixture were 10 mM cadaverine, 10 μM Amplex UltraRed (Life Technologies, Carlsbad, CA), and 1 U/mL horse radish peroxidase (Amresco, Solon, OH). 

The enzyme activity, which is proportional to the rate of increase in fluorescence, was measured for each experiment. Residual activity was calculated by dividing each rate by a maximum rate determined in the absence of inhibitor such that [residual activity] = ([rate with inhibitor] – [baseline rate])/([maximum rate] – [baseline rate]). Baseline rates, which were typically <2% of the maximum rates, were determined in the absence of enzyme and inhibitor. The residual activities from replicate experiments were averaged. For each inhibitor concentration, log([average residual activity]) was plotted against inhibition time. Linear lines of best fit forced through the origin were calculated for each data set using Graphpad Prism 5.0b (La Jolla, CA). For each inhibitor, the slopes of these lines (min^−1^) were plotted as –1/slope against 1/[inhibitor concentration] to produce a Kitz–Wilson plot. Lines of best fit (linear regression), slopes, y-intercepts, and their associated standard errors were calculated using Graphpad Prism 5.0b. The kinetic parameters *K*_I _^−^_ _^1^ and *k*_2_ were calculated such that *k*_2_ (min^−^ ^1^) = 1/(y-intercept) and *K*_I _^−^_ _^1^ (μM) = slope/(y intercept). Results are tabulated in the Supporting Information.

### Organic synthesis

General methods are described in the Supporting Information.

**Carbamate 8**. *Tert*-butyl carbazate (**7**, 300 mg, 2.27 mmol, 1 equiv), hexanoic acid (**6**, 283 μL, 2.27 mmol, 1 equiv), EDC hydrochloride (430 mg, 2.24 mmol, 1 equiv), HOBt hydrate (347 mg, 2.27 mmol, 1 equiv), and DCM (11.3 mL, 0.2 M) were added into a flask. The mixture was stirred (16 h), diluted with DCM (20 mL), washed with NaHCO_3_ (15 mL), sodium chloride (50% saturated aqueous, 15 mL), and NaHSO_4_ (10% aqueous, 15 mL), and dried with sodium sulfate. Volatiles were removed under reduced pressure, and the crude product was purified by column chromatography (40 mL silica gel, 2:1 hexane/EtOAc) to yield pure carbamate **8** (colorless oil, 271 mg, 1.18 mmol, 52% yield). **^1^H NMR** (CDCl_3_, 200 MHz) *δ* 7.60 (*s*, 1H, NH), 6.64 (*s*, 1H, NH), 2.20 (*t*, *J* = 7.3 Hz, 2H, H_2_C–C = O), 1.76–1.57 (*m*, 2H, CH_2_), 1.46 (*s*, 9H, Boc), 1.39–1.20 (*m*, 4H, 2CH_2_), (*t*, *J* = 7.4, 3H, CH_3_). ^13^**C NMR** (CDCl_3_, 50 MHz) *δ* 172.74, 155.68, 81.79, 34.01, 31.32, 28.10, 24.91, 22.30, 13.89. **IR** (neat, cm^−^ ^1^): 3314, 3255, 2968, 2934, 2875, 1738, 1673, 1647, 1494, 1365, 1239, 1158. **TLC** (2:1) hexane/EtOAc, permanganate, *R_f_* = 0.31. **MS** calculated for [C_11_H_22_N_2_O_3_Na]^+^, requires *m/z* = 253.15, found *m/z* = 253.2 (ESI).

**Hydrazide 3 hydrochloride**. Boc carbazate **8** (68 mg, 0.30 mmol, 1 equiv) was dissolved in dioxane (0.5 mL) and diluted with a solution of hydrochloric acid (8 M in dioxane, 1 mL). The flask was capped with a rubber septum, and the mixture was stirred (19 h). Volatiles were removed in a stream of nitrogen gas and then under reduced pressure to yield pure acyl hydrazide **3** hydrochloride (32 mg, 0.19 mmol, 65% yield). **^1^H NMR** (DMSO-*d*_6_, 200 MHz) *δ* 11.01 (*s*, 1H, NH), 10.70–10.20 (brs, 3H, NH_3_), 2.22 (*t*, *J* = 7.2 Hz, 2H, CH_2_–C = O), 1.53 (pent, *J* = 7.1 Hz, 2H, CH_2_), 1.36–1.11 (*m*, 4H, 2CH_2_), 0.86 (*t*, *J* = 6.5 Hz, 3H, CH_3_). ^13^**C NMR** (DMSO-*d*_6_, 50 MHz) *δ* 171.72, 32.86, 30.72, 24.45, 21.90, 13.95. **IR** (neat, cm^−^ ^1^): 3383, 2957, 2930, 2892, 1699, 1526, 1367, 1222, 1185. **MS** calculated for [C_6_H_14_N_2_OH]^+^ requires *m/z* = 131.12, found *m/z* = 131.2 (ESI).

**Carbamate 11**. A flame-dried flask was capped with a rubber septum and maintained under a nitrogen atmosphere. Into the flask were added bis(Boc)hydrazine (**10**, 400 mg, 1.71 mmol, 1.05 equiv), DMF (8 mL, 0.2 M), 1-bromoheptane (**9**, 0.240 mL, 1.63 mmol, 1 equiv), and cesium carbonate (1.11 g, 3.41 mmol, 2.1 equiv). The reaction was stirred under a nitrogen atmosphere (19 h), diluted with brine (20 mL), extracted with EtOAc (2 × 20 mL), and dried with sodium sulfate. Volatiles were removed under reduced pressure, and the crude material was purified by column chromatography (15 mL silica gel, 10:5:1 hexane/DCM/Et_2_O) to yield pure carbamate **11** (0.27 g, 0.68 mmol, 42% yield, colorless oil). Based on the NMR signals, this compound may exist as interconverting rotational isomers (that coalesce after the Boc groups are removed). **^1^H NMR** (CDCl_3_, 200 MHz) *δ* 6.38–6.01 (brm, 1H, NH), 3.41 (*t*, *J* = 7.4 Hz, 2H, H_2_C–N), 1.61–1.49 (*m*, 2H, CH_2_), 1.46 (*s*, 9H, Boc), 1.45 (*s*, 9H, Boc), 1.36–1.19 (*m*, 8H, 4CH_2_), 0.87 (*t*, *J* = 7.1 Hz, 3H, CH_3_). ^13^**C NMR** (CDCl_3_, 50 MHz) *δ* 155.30, 80.76, 49.42 (brm), 31.72, 28.93, 28.13, 27.41, 26.59, 22.51, 13.98. **IR** (neat, cm^−1^): 3278, 2979, 2920, 2857, 1698, 1514, 1404, 1364, 1287, 1148, 756. **TLC** (4:1) hexane/EtOAc, permanganate, *R_f_* = 0.63. **MS** calculated for [C_17_H_34_N_2_O_4_Na]^+ ^ requires *m/z* = 353.24, found *m/z* 353.3 (ESI).

**Hydrazine 4 Trifluoroacetate**. Carbamate **11** (16.5 mg, 0.050 mmol 1 equiv), DCM (1.5 mL), and trifluoroacetic acid (0.5 mL) were added into a vial. The reaction was stirred (6 h), and volatiles were removed under reduced pressure (toluene azeotrope) to yield pure hydrazine **4** trifluoroacetate (9.4 mg, 0.038 mmol, 77% yield, colorless oil). **^1^H NMR** (DMSO-*d*_6_, 200 MHz) *δ* 2.88 (*t*, *J* = 7.9 Hz, 2H, CH_2_–N), 1.64–1.42 (*m*, 2H, H_2_C), 1.31–1.11 (*m*, 8H, 8CH_2_), 0.86 (*t*, *J* = 6.5 Hz, 3H, CH_3_). ^13^**C NMR** (DMSO-*d*_6_, 50 MHz) *δ* 158.89 (*q*, *J* = 31.9 Hz, O = C–CF_3_), 116.94 (*q*, *J* = 297.0 Hz, CF_3_), 50.63, 31.13, 28.37, 26.04, 24.66, 22.09, 13.96. **IR** (neat cm^−^ ^1^): 2957, 2928, 2859, 1684, 1668, 1203, 1140. **MS** calculated for [C_7_H_18_N_2_H]^+^, requires *m/z* = 131.15, found *m/z*** **= 131.2 (ESI).

**Boc-Semicarbazide 14**. *n*-Butylamine (0.550 mL, 5.56 mmol, 1 equiv), DCM (10 mL), and a sodium bicarbonate solution (saturated aqueous, 10 mL) were added into a flask. The mixture was cooled in an ice bath (0 °C). Triphosgene (550 mg, 1.86 mmol, 0.33 equiv) and dry DCM (5 mL) were added into a flask. The solution was cooled to 0 °C and then added into the amine solution. The mixture was stirred (30 min) and then allowed to settle. The organic phase was isolated, dried with sodium sulfate, and returned to the ice bath. *tert*-Butyl carbazate (**7**, 736 mg, 5.57 mmol, 1 equiv) was added. The mixture was stirred (ice bath, 15 min, then ambient temperature, 15 h), washed with sodium bicarbonate (saturated aqueous, 15 mL) and sodium bisulfate (1 M, 15 mL), and dried with sodium sulfate. Volatiles were removed under reduced pressure, and the crude material was purified by column chromatography (20 mL silica gel, 2:1 DCM/EtOAc) to yield pure Boc-semicarbazide **14** (301 mg, 1.30 mmol, 23%, colorless oil). Based on the ^13^C NMR, this compound appears to exist as a mixture of interconverting rotational isomers (coalesce after the Boc group is removed). **^1^H NMR (**CDCl_3_, 200 MHz) *δ* 6.32 (*s*, 1H, NH), 6.21 (*s*, 1H, NH), 5.40–5.23 (brs, 1H, NH), 3.24 (*q*, *J* = 6.9 Hz, 2H, CH_2_–N), 1.66–1.23 (*m*, 4H, 2CH_2_), 1.48 (*s*, 9H, Boc), 0.91 (*t*, *J* = 7.0 Hz, 3H, CH_3_). ^13^**C NMR (**CDCl_3_, 50 MHz, major signals) *δ* 159.22, 156.55, 81.38, 39.57, 31.97, 27.99, 19.76, 13.63. **IR** (neat, cm^−1^): 3315, 2962, 2933, 1721, 1661, 1552, 1368, 1244, 1158, 909, 731. **MS** calculated for [C_10_H_21_N_3_O_3_Na]^+^ requires *m/z* = 254.15, found *m/z* = 254.1 (ESI). **TLC** (2:1) DCM/EtOAc, permanganate, *R_f_* = 0.30.

**Semicarbazide 5 Hydrochloride**. Boc-semicarbazide **14** (68.2 mg, 0.295 mmol, 1 equiv), dioxane (0.7 mL) and a solution of hydrogen chloride (8 M in dioxane, 1 mL) were added into a vial. The vial was capped with a rubber septum, and the mixture was stirred (5 h). Volatiles were removed in a stream of nitrogen gas and then under reduced pressure to yield pure semicarbazide **5** hydrochloride (47.8 mg, 0.285 mmol, 97% yield, white solid). **^1^H NMR** (DMSO-d_6_, 200 MHz) *δ* 9.98–9.63 (brs, 3H, NH_3_), 8.63 (*s*, 1H, NH), 7.02 (*t*, *J* = 5.8 Hz, 1H, NH–C), 3.04 (*q*, *J* = 6.1 Hz, 2H, CH_2_–N), 1.47–1.18 (*m*, 4H, 2CH_2_), 0.86 (*t*, *J* = 7.3, 3H, CH_3_). ^13^**C NMR** (DMSO-*d*_6_, 50 MHz) *δ* 157.27, 31.70, 19.47, 13.76. (One signal overlaps the solvent signal). **IR** (neat, cm^−^ ^1^): 3299, 2862, 2663, 1682, 1557, 1493, 1282, 647. **MS** calculated for [C_5_H_13_N_3_OH]^+^ requires *m/z* = 132.11, found *m/z* = 132.2 (ESI).

## Results

**Scheme 1.**
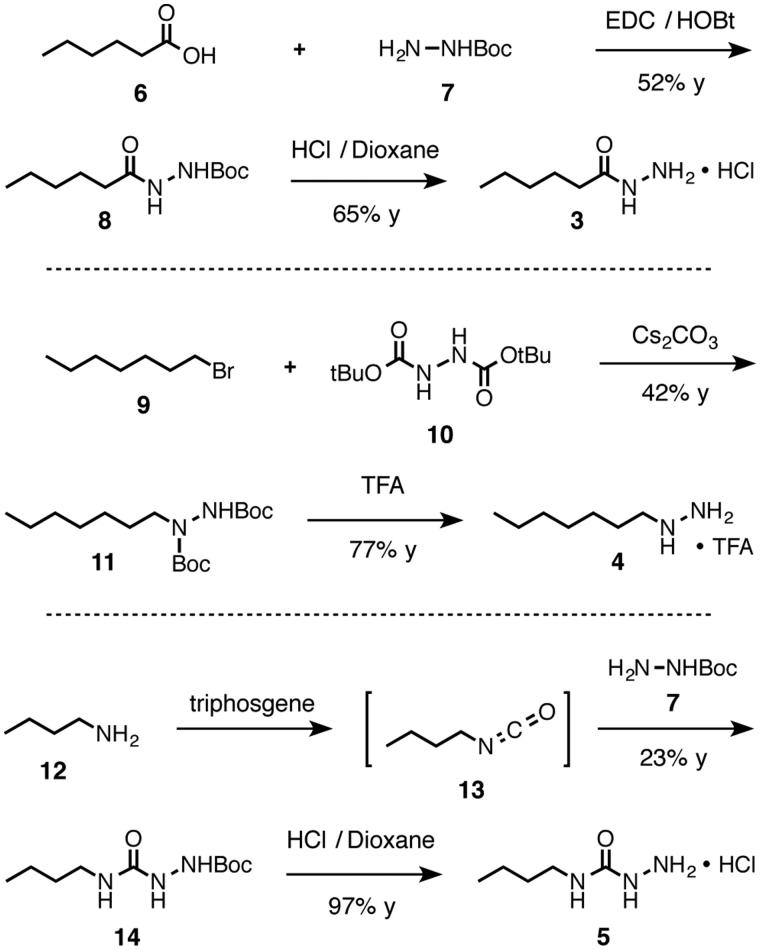
Synthesis of inhibitors **3**, **4**, and **5**. EDC = *N*-(3-dimethylaminopropyl)-*N*'-ethylcarbodiimide hydrochloride; HOBt = hydroxybenzotriazole hydrate; TFA = trifluoroacetic acid; Boc = *tert*-butoxycarbonyl; y = yield.

Compounds **3**–**5**, which are representative members of the hydrazide, alkyl hydrazine, and semicarbazide families, each connect a hydrazine-containing core functional group to a simple aliphatic chain (Figure 2). To synthesize these compounds, we developed synthetic strategies (Scheme 1) that can avoid contamination from free hydrazine (H_2_N–NH_2_), which is a potent inhibitor itself. Hydrazide **3** was synthesized by a carbodiimide-promoted amide bond forming reaction between hexanoic acid^6^ and mono(Boc)-protected hydrazine **7**. Alkyl hydrazine **4** was formed by alkylating bis(Boc)hydrazine **10** under basic conditions. And finally, semicarbazide **5** was synthesized by trapping isocyanate **13** with mono(Boc)-protected hydrazine **7**.

**Figure 3.**
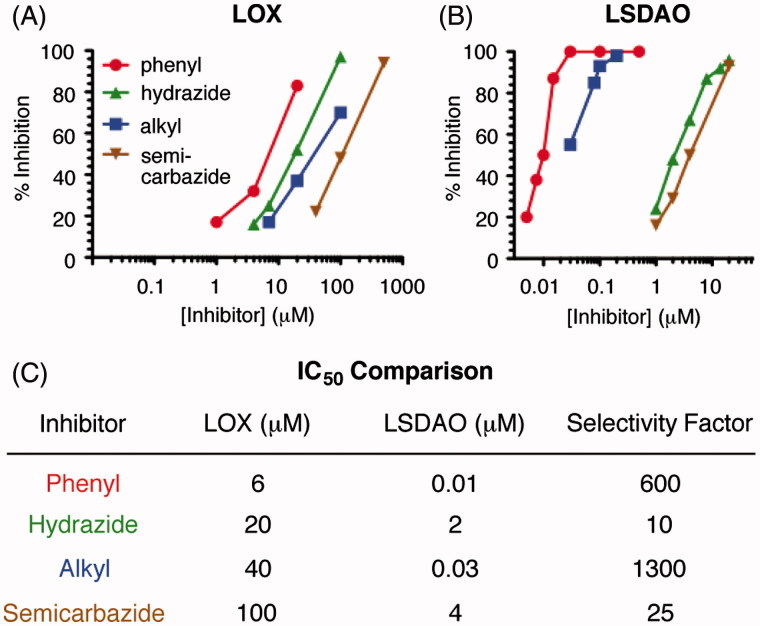
Inhibition of amine oxidases with hydrazine inhibitors. (A) Inhibition of LOX with a 10-min preincubation. (B) Inhibition of LSDAO with a 10-min preinhibition. (C) Comparison of the IC_50_ values for inhibiting LOX and LSDAO. Selectivity factor is calculated as the LOX IC_50_ value divided by the LSDAO IC_50_ value. phenyl = phenylhydrazine (1); hydrazide = **3**; alkyl = alkyl hydrazine **4**; semicarbazide = **5**.

With these three synthetic hydrazine inhibitors in hand, we used an activity assay to compare these compounds to phenylhydrazine as inhibitors of LOX and LSDAO. After preincubating the enzyme with an inhibitor, the amount of active enzyme remaining was measured by a previously reported activity assay^35^ that uses horseradish peroxidase and Amplex red to generate a fluorescence readout proportional to the rate at which active enzyme produces its hydrogen peroxide by-product. Comparing the amount of LOX inhibition after a 10-min incubation time (Figure 3(A)) shows that phenylhydrazine (IC_50 _=_ _6 μM) is somewhat more potent than the other three hydrazine-based inhibitors (IC_50 _=_ _20–100 μM), although the four IC_50_ values only span a 17-fold range. Of the three new inhibitors, hydrazide **3** is the most potent, followed by alkyl hydrazine **4** and then by semicarbazide **5**. In contrast, the inhibitions of LSDAO (Figure 3(B)) show a significantly broader range of potencies. While the IC_50_ values of hydrazide inhibitor **3** and semicarbazide inhibitor **5** are in the micromolar range, 2 and 4 μM, respectively, the values for alkyl hydrazine **4** and phenylhydrazine are substantially less, 30 and 10 nM, respectively. To quantify the selectivity of each inhibitor, we defined a numerical “selectivity factor” as the inhibitor's IC_50_ value for LOX divided by its IC_50_ value for LSDAO (Figure 3(C)). While each of the four compounds inhibits LSDAO at lower concentration than LOX, the degree of this selectivity varies considerably. While the selectivity factors of phenylhydrazine and alkyl hydrazine **4**, 600 and 1300, respectively, show a strong preference for LSDAO, the selectivity factors of semicarbazide **5** and hydrazide **3** are much lower, only 25 and 10, respectively.

**Figure 4.**
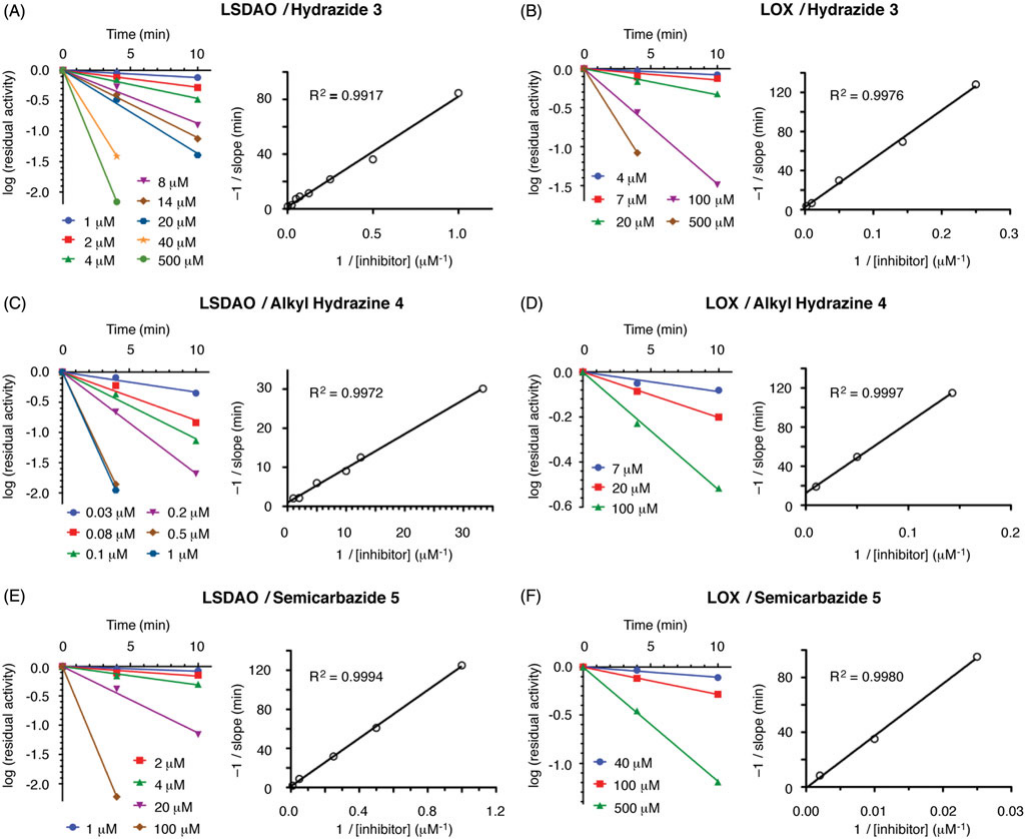
Kinetic analysis of inhibitors **3**, **4**, and **5** inhibiting LSDAO and LOX. −1/slope = −1/[slope of the log(residual activity) against time line]; time = preincubation time of enzyme with inhibitor before analysis.

**Figure 5.**
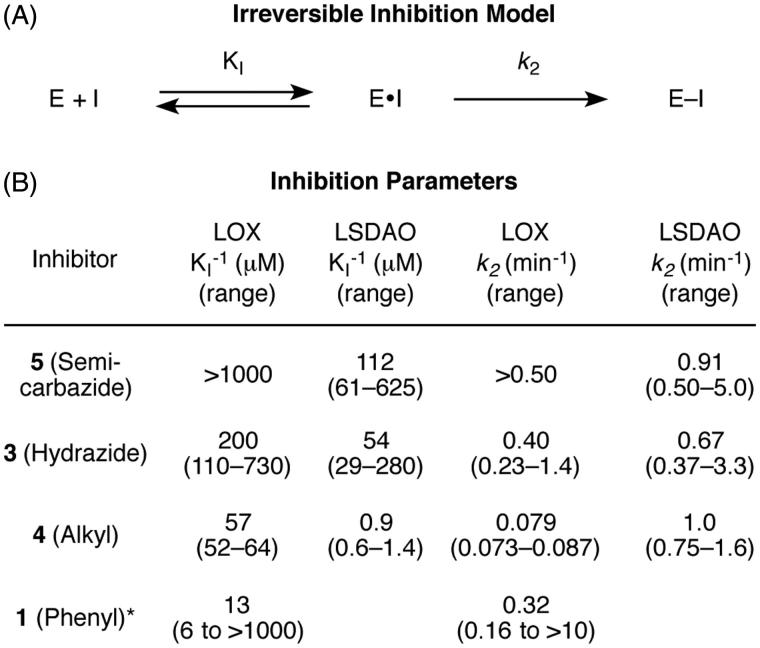
Irreversible kinetics of hydrazine inhibitors on amine oxidases. A. The model used for analysis. *K*_I_ is the equilibrium constant for the reversible association. *k_2_* is the first-order rate constant for the irreversible reaction. B. Comparison of kinetic parameters. *Phenyl hydrazine data is from reference 25. Error ranges were calculated from the standard errors of the slope and y-intercept values.

To confirm that the three synthesized hydrazines act as irreversible inhibitors and to measure the kinetic parameters of binding for each, we expanded the conditions of our assay to measure the amount of inhibition over time. For both LOX and LSDAO, all three inhibitors produce more inhibition with longer preincubation times (Figure 4) in a manner that is consistent with a model of irreversible inhibition involving equilibrium binding of the inhibitor followed by first-order irreversible inactivation (Figure 5(A)). In each case, plotting the logarithm of the percent of active enzyme remaining against time gives linear functions that pass through the origin for each inhibitor concentration. Analysis by the classic method of Kitz and Wilson^36^, based on the mechanism of Figure 5(A), shows that plotting the reciprocal of the slopes of each of these lines against the reciprocal of the inhibitor concentrations gives straight lines for each. From the slopes and y-intercepts of these lines, the kinetic parameters *K*_I_ (the reversible binding constant) and *k_2_* (the rate constant for irreversible reaction) (Figure 5(A)) can be calculated^36^.

The results from the kinetics analyses of the three synthetic hydrazines as irreversible inhibitors of LOX and LSDAO enable specific comparisons of their kinetic parameters to be made. For the inhibition of LOX with phenylhydrazine, data previously reported by Kagan and coworkers are also included in this comparison^25^ but reprocessed using the same Kitz–Wilson analysis and error calculations that were used for the new data sets (Supplementary Figure S1). Comparing the reversible affinities of the inhibitors for the enzymes (*K*_I_) shows two major trends (Figure 5(B)). First, each of the new inhibitors has higher affinity for LSDAO than for LOX, which is consistent with the fact that each of the inhibitors inhibits LSDAO more potently overall and which illustrates the importance of the reversible binding of the inhibitor for enzyme selectivity. Second, the affinities of the four inhibitors for LOX vary substantially. Semicarbazide **5** has only minimal affinity, whereas hydrazide **3**, alkyl hydrazine **4**, and phenylhydrazine have affinities in the high, middle, and low micromolar ranges, respectively, (although the error range on the phenylhydrazine data is quite large due to the mathematical issues of dividing by numbers near zero.) It may be that the inhibitors with additional polar and hydrogen-bonding groups near their hydrazine unit are less well matched to a hydrophobic active site, especially for LSDAO, which is known to have its active site buried in a hydrophobic cleft^37^.

Comparing the rate constants for the irreversible step, which are calculated as the reciprocal of the y-intercept, shows that most of the values are at least 0.2 min^−1^ and have overlapping error ranges (Figure 5(B)). Some of the error ranges for the y-intercepts are near or touching zero (see the Supporting Information for values), which causes the error range of the rate constants to become quite large after the reciprocal function is applied; this amplification of error is a known limitation of Kitz–Wilson analysis^38^. Nevertheless, the only inhibitor–enzyme combination whose rate constant for the irreversible step is less than 0.2 min^−1^ is the inhibition of LOX with alkyl inhibitor **4**, whose rate constant is in the 0.07–0.09 min^−1^ range. This difference in the relative reaction rates for LOX correlates with the relative basicities of the inhibitors and may result from a difference in the protonation equilibria of the four amino groups; at neutral pH, alkyl hydrazines (p*K*_a_ of conjugate acids = 8–9) will have a larger percent of their molecules protonated and therefore unable to react compared to phenylhydrazine (p*K*_a_ of conjugate acid = 5), hydrazides (p*K*_a_ of conjugate acids = 3–4), or semicarbazides (p*K*_a_ of conjugate acids = 3–4). For the reactions with LSDAO, the active site – which is believed to be located in a hydrophobic channel^37^ – may modulate these p*K*_a_ values and negate this difference by only allowing nonprotonated inhibitors into the active site.

## Discussion

BAPN is generally considered to be the most potent and selective inhibitor for LOX. Its selectivity is believed to relate to the fact that it likely modifies a specific (currently unidentified) active-site residue near the lysyl tyrosylquinone that is not present in other amine oxidases^27^. An IC_50_ value of 0.2 μM and full inhibition at 10 μM have been reported for a 2-h incubation^27^. These data were fit to a Kitz–Wilson plot to give *K*_I_ = 6 μM and *k_2 _*=*_ _*0.16 min^−1^ (pH 8.0 and 37 °C), although the authors note that the data clearly diverge from linearity at later time points, which is perhaps due to the more complex mechanism of inhibition.

Previous reports have utilized phenylhydrazine to inhibit LOX; however, much less is known about other families of hydrazine-containing organic compounds as LOX inhibitors. For hydrazides, it has been reported that the hydrazide of isonicotinic acid can partially inhibit LOX in chick embryos *in vivo*^39^. For semicarbazide, it has been reported that semicarbazide itself (just H_2_NC(O)NHNH_2_ with no organic scaffold) is nearly as effective as BAPN at inhibiting rat lysyl oxidase *in vivo*^40^. *In vitro* experiments showed an IC_50_ value of 30 μM (inhibition time not specified) for semicarbazide^41^. The new data presented herein allow for direct comparisons of alkyl hydrazines, hydrazides, and semicarbazides to phenylhydrazine under identical conditions. With a 10-min incubation time, acyl inhibitor **3** and alkyl inhibitor **4** have IC_50_ values of 20 and 40 μM, respectively, which are not too different from that measured for phenylhydrazine, 6 μM, or from the other hydrazine inhibitors mentioned above. Semicarbazide **5**, which has an IC_50_ of 100 μM, does not perform as well as the other compounds, perhaps due to the extra N–H unit being poorly matched to a hydrophobic region of the enzyme's active site. While these new inhibitors are not as potent or selective as BAPN, due to their straightforward mechanisms of inhibition, the ease with which they can be incorporated into larger molecules, and the precedent of hydrazine groups in FDA-approved drugs, we are optimistic that they have the potential to be able to be developed into inhibitors with strong potency and high selectivity.

The comparisons between LOX and LSDAO presented herein provide useful insights into how these simple organic hydrazine structures could be applied as warhead groups in larger molecules designed to selectively target LOX. Although phenylhydrazine is the most potent of the four hydrazine compounds at inhibiting LOX, hydrazide **3** shows properties that may be more advantageous. Using LSDAO as an example “off-target” enzyme suggests that phenylhydrazine, whose IC_50_ value for LSDAO is 600-fold more potent than that for LOX, may be difficult to develop into an inhibitor that is selective for LOX. Hydrazide **3**, which has only a 10-fold difference between the two enzymes, might be a better place to start, even though it has 3-fold lower affinity for LOX than phenylhydrazine does.

The kinetics analysis of hydrazide **3** also suggests that the hydrazide core will be an excellent place to begin developing inhibitors that selectively target LOX. The rate constants for hydrazide **3** to bind LOX and LSDAO (0.4 and 0.7 min^−1^ with overlapping error bars) are similar to those seen for some FDA-approved covalent inhibitors such as the anticancer drug Afatinib (*k_2 _*=*_ _*0.14 min^−1^)^42^. It is expected that this rate constant will remain relatively constant as the scaffold around the hydrazide core is changed because the reactivity after it binds to the active site is a property of the core reactive group, not of the scaffold. The reversible affinities of this molecule (200 and 50 μM with overlapping error bars for LOX and LSDAO) are weak enough that they should be able to be improved by building this core reactive group into a molecular structure that is a better match to the shape of LOX's-active site. Stabilizing interactions, such as matched hydrogen-bonding groups or complementary hydrophobic interactions, between the inhibitor's scaffold and the enzyme should increase its affinity. Indeed, similar structure–activity relationships have been described for hydrazine-containing molecules inhibiting other enzymes^43^. Additionally, as the scaffold becomes more elaborate and well matched to LOX's active site, it is likely that the structure will become a poorer match with off-target enzymes, many of which have narrow substrate-binding channels, due to steric repulsions or mismatched polar interactions. Together, these structural relations will increase the inhibitor's selectivity by simultaneously increasing its reversibly affinity for LOX and lowering its affinity for other enzymes.

In conclusion, the data reported herein compares four families of hydrazine-based inhibitors for their abilities to inhibit quinone-dependent amine oxidases. Representative members of the hydrazide, alkyl hydrazine, and semicarbazide families were synthesized and compared to phenylhydrazine as inhibitors of both LOX and LSDAO. Phenylhydrazine and the alkyl hydrazine inhibit LOX but are much more potent inhibitors of LSDAO. The hydrazide and semicarbazide inhibitors are much less potent inhibitors of LSDAO suggesting that they have more promise for development as selective inhibitors of LOX. Of these two, the hydrazide is five times as potent an inhibitor of LOX and has only a slight preference for LSDAO, which makes it a more promising core functionality to develop into inhibitors that selectively target lysyl oxidase.

## Supplementary Material

IENZ_1265518_Supplementary_Material.pdf
