# Supplementary material for: Comparing hydrazine-derived reactive groups as inhibitors of quinone-dependent amine oxidases
Source: J Enzyme Inhib Med Chem. 2017 Jan 23;32(1):496–503. doi: 10.1080/14756366.2016.1265518 (PMC6009937; doi:10.1080/14756366.2016.1265518)

Supporting Information

Comparing hydrazine-derived reactive groups as inhibitors of quinone-dependent amine oxidases

Ashley A. Burke, Elizabeth S. Severson, Shreya Mool, Maria J. Solares Bucaro,
Frederick T. Greenaway,* Charles E. Jakobsche*

Carlson School of Chemistry & Biochemistry, Clark University, Worcester, MA

Email: cjakobsche@clarku.edu

Table of Contents

1. Analysis of Previous Data S2
2. Results of Inhibition Assay S3
3. General Synthetic Procedures S5
4. NMR Spectra of Synthesized Compounds S6

1. Analysis of Previous Data

The kinetics of inhibition of LOX with phenyl hydrazine has been previously studied by Williamson and coworkers (1). Their data (from their Figure 3A) was analyzed using the Kitz–Wilson method that we have used to analyze our own data. (The Y axis was converted to log(% activity remaining), the slopes were measured, 1/slope vs 1/[inhibitor] was plotted, and a line of best fit was calculated with Prism, Supplementary Figure S1).

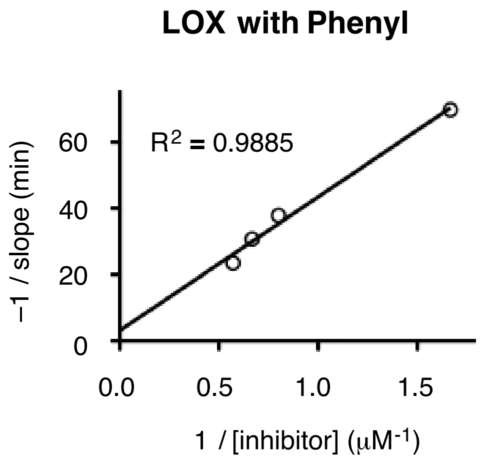

Supplementary Figure S1. Kitz–Wilson analysis of data from Williamson and coworkers for phenyl hydrazine inhibiting LOX.

Kinetics Parameters for Inhibiting LOX with Phenyl Inhibitor 1

Kitz–Wilson Y-Intercept = 3.1 +/– 3.1 min
Kitz–Wilson slope = 40 +/– 3 min • μM
k_2 = 0.32 min^{-1} (error range > 0.16 min^{-1})
KI^{-1} = 13 μM (error range > 6 μM)

[1] Williamson P R, Kittler J M, Thanassi J W, Kagan H M. Reactivity of a functional carbonyl moiety in bovine aortic lysyl oxidase. Biochem. J. 1986;235:597–605.

2. Results of Inhibition Assay

Average Percent Activity Remaining for Inhibiting LSDAO with Phenyl Inhibitor 1

Minutes	0.005 μM	0.0075 μM	0.010 μM	0.015 μM	0.03 μM	0.1 μM	0.5 μM
10	80	62	50	13	0	0	0

Average Percent Activity Remaining for Inhibiting LOX with Phenyl Inhibitor 1

Minutes	1 μM	2 μM	20 μM
10	83	73	17

Average Percent Activity Remaining for Inhibiting LSDAO with Hydrazide Inhibitor 3

Minutes	1 μM	2 μM	4 μM	8 μM	14 μM	20 μM	40 μM	500 μM
4	90	80	71	54	40	33	4	0.7
10	76	52	33	13	8	4		

Kinetics Parameters for Inhibiting LSDAO with Hydrazide Inhibitor 3

Kitz–Wilson Y-Intercept = 1.5 +/– 1.2 min
Kitz–Wilson slope = 81 +/– 3 min • μM
k_2 = 0.67 min^{-1} (error range = 0.37–3.3 min^{-1})
K_I^{-1} = 54 μM (error range = 29–280 μM)

Average Percent Activity Remaining for Inhibiting LOX with Hydrazide Inhibitor 3

Minutes	4 μM	7 μM	20 μM	100 μM	500 μM
4	93	78	69	27	8
10	84	75	48	3	

Kinetics Parameters for Inhibiting LOX with Hydrazide Inhibitor 3

Kitz–Wilson Y-Intercept = 2.5 +/– 1.8 min
Kitz–Wilson slope = 496 +/– 14 min • μM
k_2 = 0.40 min^{-1} (error range = 0.23–1.4 min^{-1})
K_I^{-1} = 200 μM (error range = 110–730 μM)

Average Percent Activity Remaining for Inhibiting LSDAO with Alkyl Inhibitor 4

Minutes	0.03 μM	0.08 μM	0.1 μM	0.2 μM	0.5 μM	1 μM
4	81	60	43	22	1	1
10	45	15	7	2		

Kinetics Parameters for Inhibiting LSDAO with Alkyl Inhibitor 4

Kitz–Wilson Y-Intercept = 1.0 +/– 0.4 min
Kitz–Wilson slope = 0.88 +/– 0.02 min • μM
k_2 = 1.0 min^{-1} (error range = 0.75–1.6 min^{-1})
K_I^{-1} = 0.9 μM (error range = 0.6–1.4 μM)

Average Percent Activity Remaining for Inhibiting LOX with Alkyl Inhibitor 4

Minutes	7 μM	20 μM	100 μM
4	89	82	59
10	83	63	30

Kinetics Parameters for Inhibiting LOX with Alkyl Inhibitor 4

Kitz–Wilson Y-Intercept = 12.6 +/– 1.1 min
Kitz–Wilson slope = 719 +/– 13 min • μM
k_2 = 0.079 min^{-1} (error range = 0.073–0.087 min^{-1})
K_I^{-1} = 57 μM (error range = 52–64 μM)

Average Percent Activity Remaining for Inhibiting LSDAO with Semicarbazide Inhibitor 5

Minutes	1 μM	2 μM	4 μM	20 μM	100 μM
4	89	78	69	41	1
10	84	71	50	7	

Kinetics Parameters for Inhibiting LSDAO with Semicarbazide Inhibitor 5

Kitz–Wilson Y-Intercept = 1.1 +/– 0.9 min
Kitz–Wilson slope = 123 +/– 2 min • μM
k_2 = 0.91 min^{-1} (error range = 0.50–5.0 min^{-1})
K_I^{-1} = 112 μM (error range = 61–625 μM)

Average Percent Activity Remaining for Inhibiting LOX with Semicarbazide Inhibitor 5

Minutes	40 μM	100 μM	500 μM
4	93	76	35
10	78	52	6

Kinetics Parameters for Inhibiting LOX with Semicarbazide Inhibitor 5

Kitz–Wilson Y-Intercept = -0.7 +/– 2.7 min
Kitz–Wilson slope = 3806 +/– 170 min • μM
k_2 = >0.50 min^{-1}
K_I^{-1} = >1000 μM

3. General Synthetic Procedures

Abbreviations

AcOH = acetic acid
DCM = dichloromethane
DMF = *N*,*N*-dimethylformamide
DMSO = dimethylsulfoxide
EDC = N-(3-dimethylaminopropyl)-N'-ethylcarbodiimide
Ether = diethyl ether
EtOAc = ethyl acetate
HOBt = hydroxybenzotriazole hydrate
MeOH = methanol
Quant = quantitative conversion
TEA = triethylamine
TFA = trifluoroacetic acid
THF = tetrahydrofuran
y = yield

General Procedures

Column chromatography was performed with 60 Å 40–63 μm silia–P flash silica gel.

Solvents for reactions (DMF, DCM, THF, and toluene) were dried using a LC Technology Solutions purification system. Other solvents were used as received unless noted otherwise.

Chemicals were purchased from Fisher, VWR, or Sigma–Aldrich and used as received unless noted otherwise.

NMR Spectra were measured in $CDCl_3$ at ambient temperature unless otherwise noted.

^{1}H NMR spectra were recorded on either a 600 or 200 MHz Varian spectrometer. Chemical shifts are reported in ppm (δ) relative to tetramethylsilane using the solvent as a reference ($CDCl_3$ = 7.26 ppm, DMSO-d_6 = 2.49 ppm, D_2O = 4.80 ppm, CD_3OD = 3.30). The following is an example data point: chemical shift (multiplicity [s = singlet, d = doublet, t = triplet, q = quartet, pent = pentet, sext = sextet, sept = septet, oct = octet, m = multiplet, br = broad, and combinations thereof], coupling constants [Hz], integration, assignment [if any]).

^{13}C NMR spectra were recorded on a 600 or 200 MHz (150 or 50 MHz) Varian spectrometer with complete proton decoupling. Chemical shifts are reported in ppm (δ) relative to tetramethylsilane using the solvent or MeOH as a reference ($CDCl_3$ = 77.16 ppm, DMSO-d_6 = 39.52 ppm, CD_3OD = 49.00 ppm, MeOH = 49.50).

IR spectra were recorded on a Perkin Elmer Spectrum 100 FT–IR spectrometer with Perkin Elmer Spectrum software. Spectra are partially reported (υ_{max}, cm^{-1}).

MS were obtained either on an Agilent Technologies 6120 quadrupole LC/MS system with an 1260 Infinity liquid chromatography system at Clark University or at The University of Illinois Urbana–Champagne's Mass Spectrometry Center.

TLC was performed on 60 Å F_{254} pre-coated silica gel plates. Samples were visualized by either ultraviolet irradiation, potassium permanganate staining, or cerium ammonium molybdate staining.

Optical Rotations were obtained with a Rudolph Research Autopol II automatic polarimeter.

Yield refers to isolated material.

Quantitative recovery means that mostly pure material was recovered in approximately the expected mass, and the material was used directly for the next step without purification.

4. NMR Spectra of Synthesized Compounds

^{1}H NMR Spectrum of 3

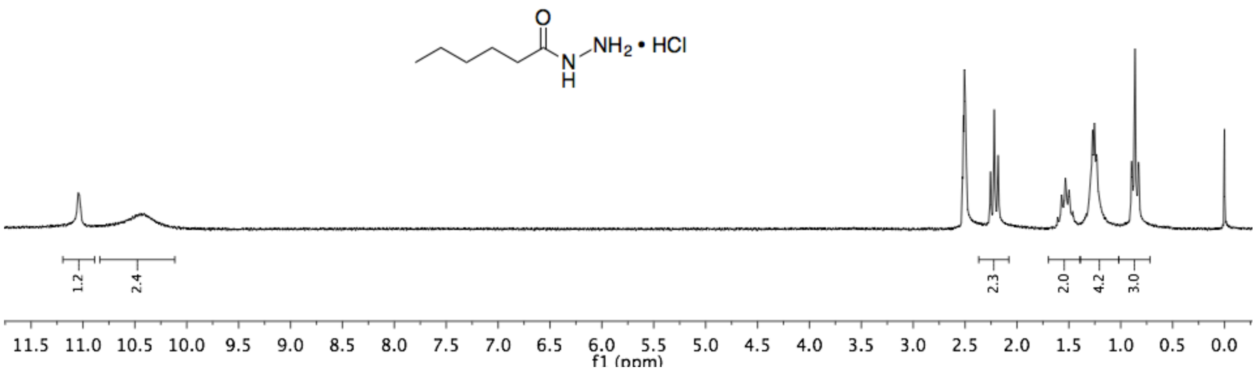

^{13}C NMR Spectrum of 3

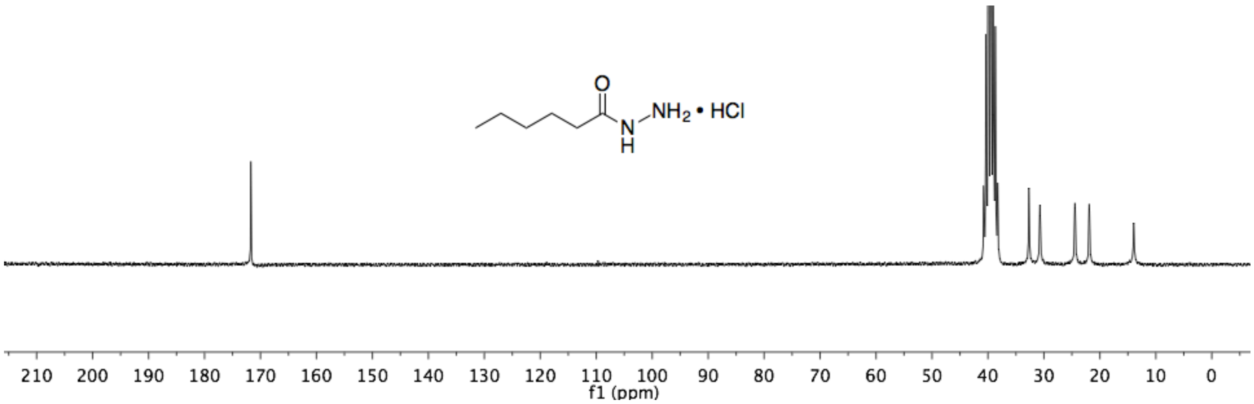

^{1}H NMR Spectrum of 4

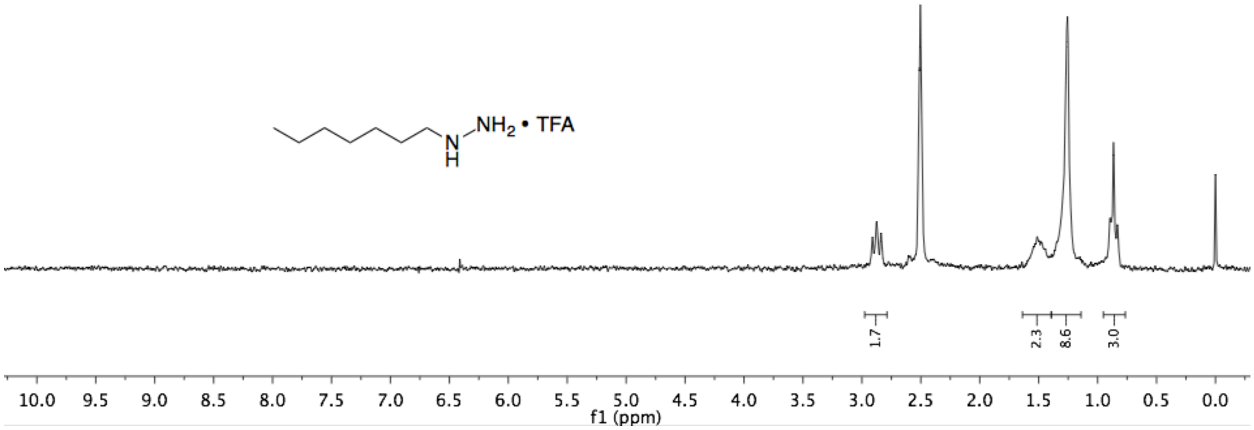

^{13}C NMR Spectrum of 4

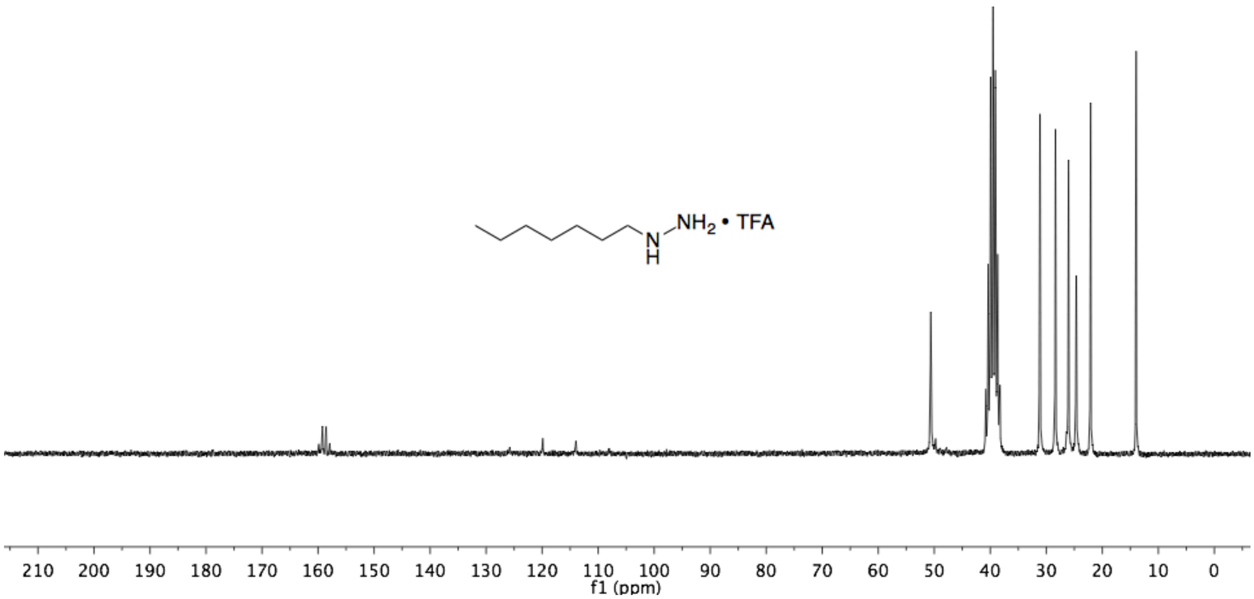

^{1}H NMR Spectrum of 5

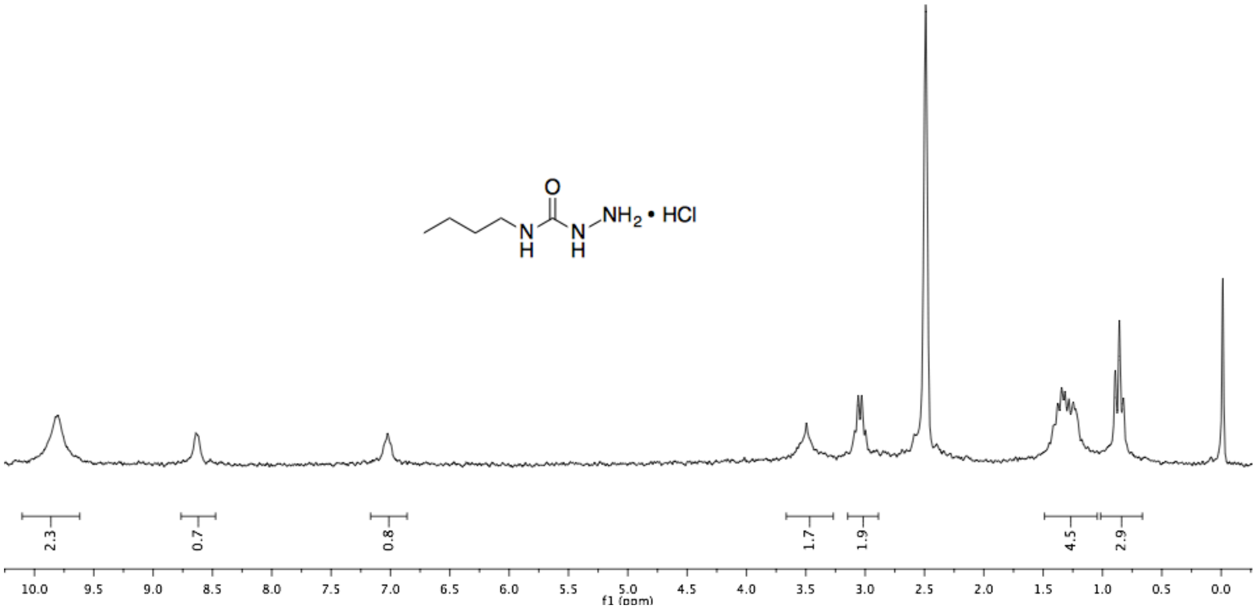

^{13}C NMR Spectrum of 5

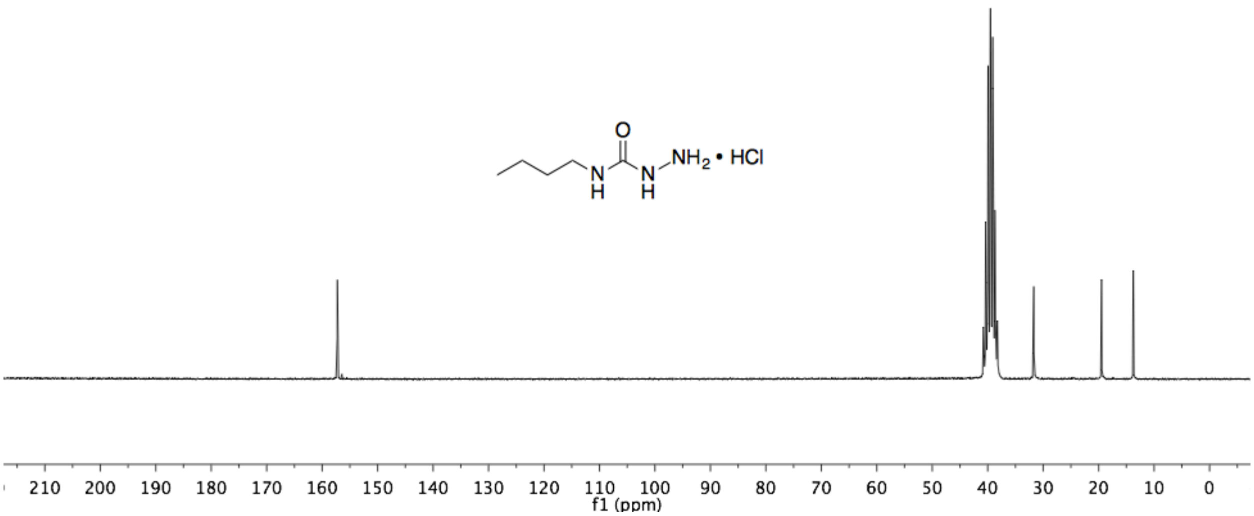

^{1}H NMR Spectrum of 8

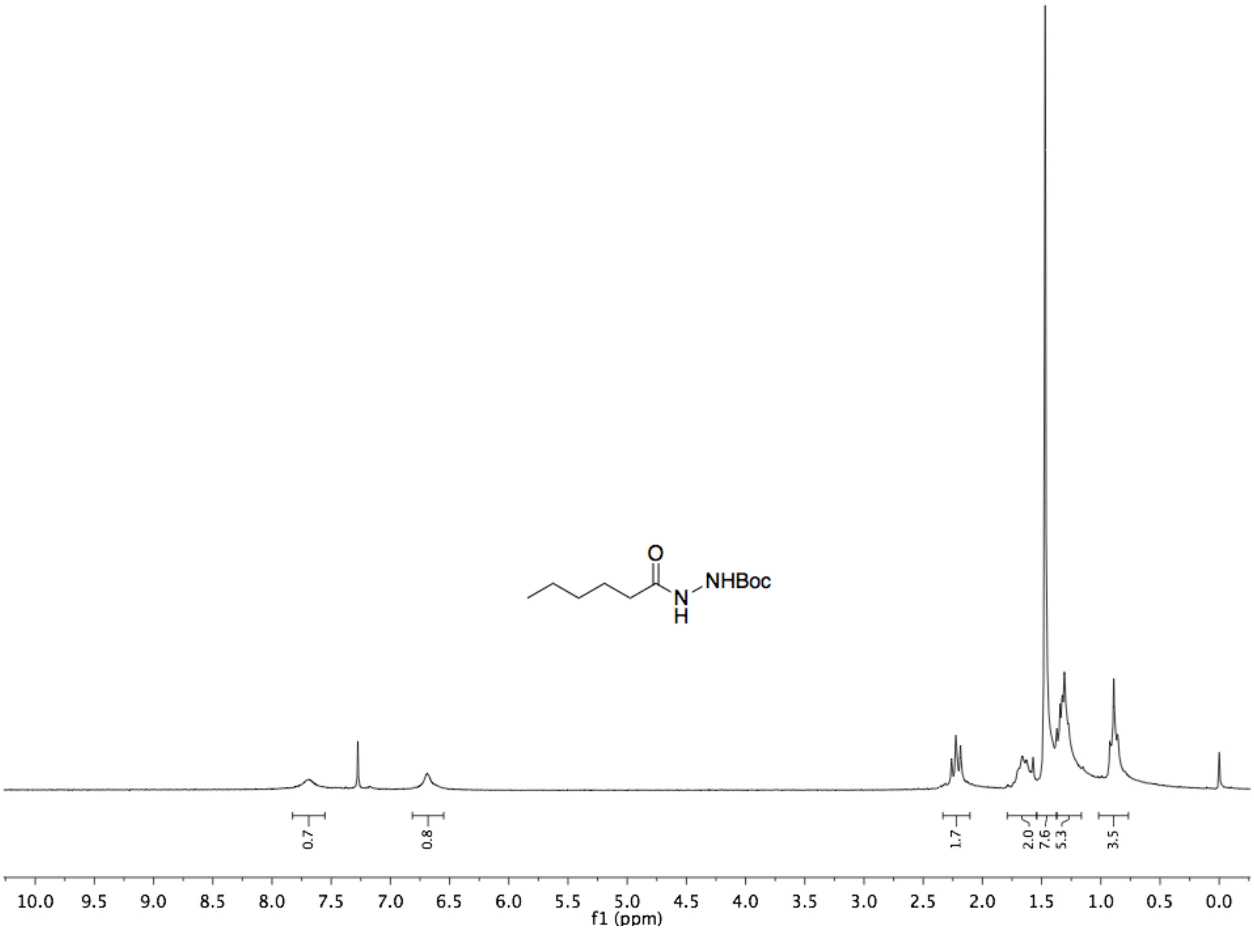

^{13}C NMR Spectrum of 8

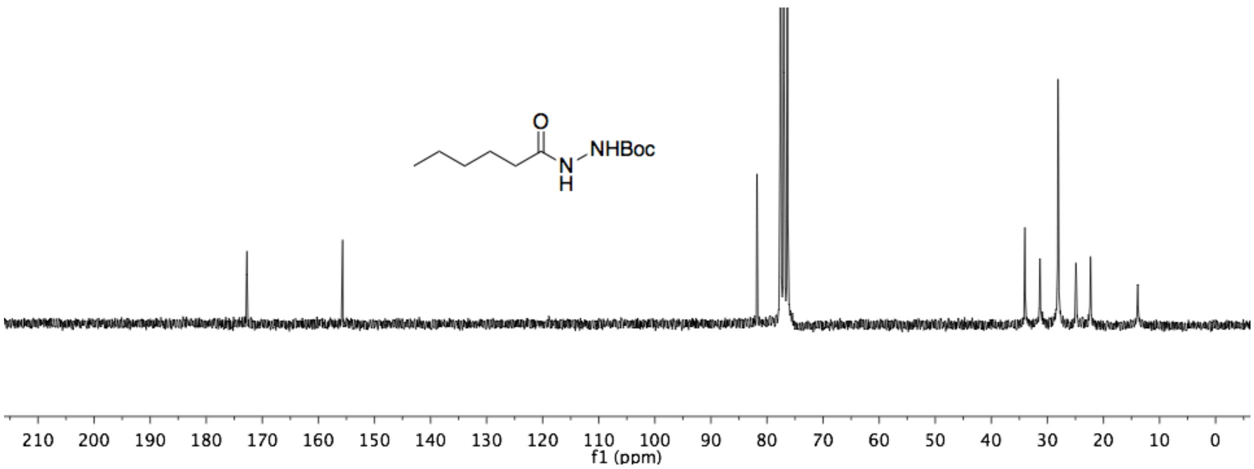

^{1}H NMR Spectrum of 10

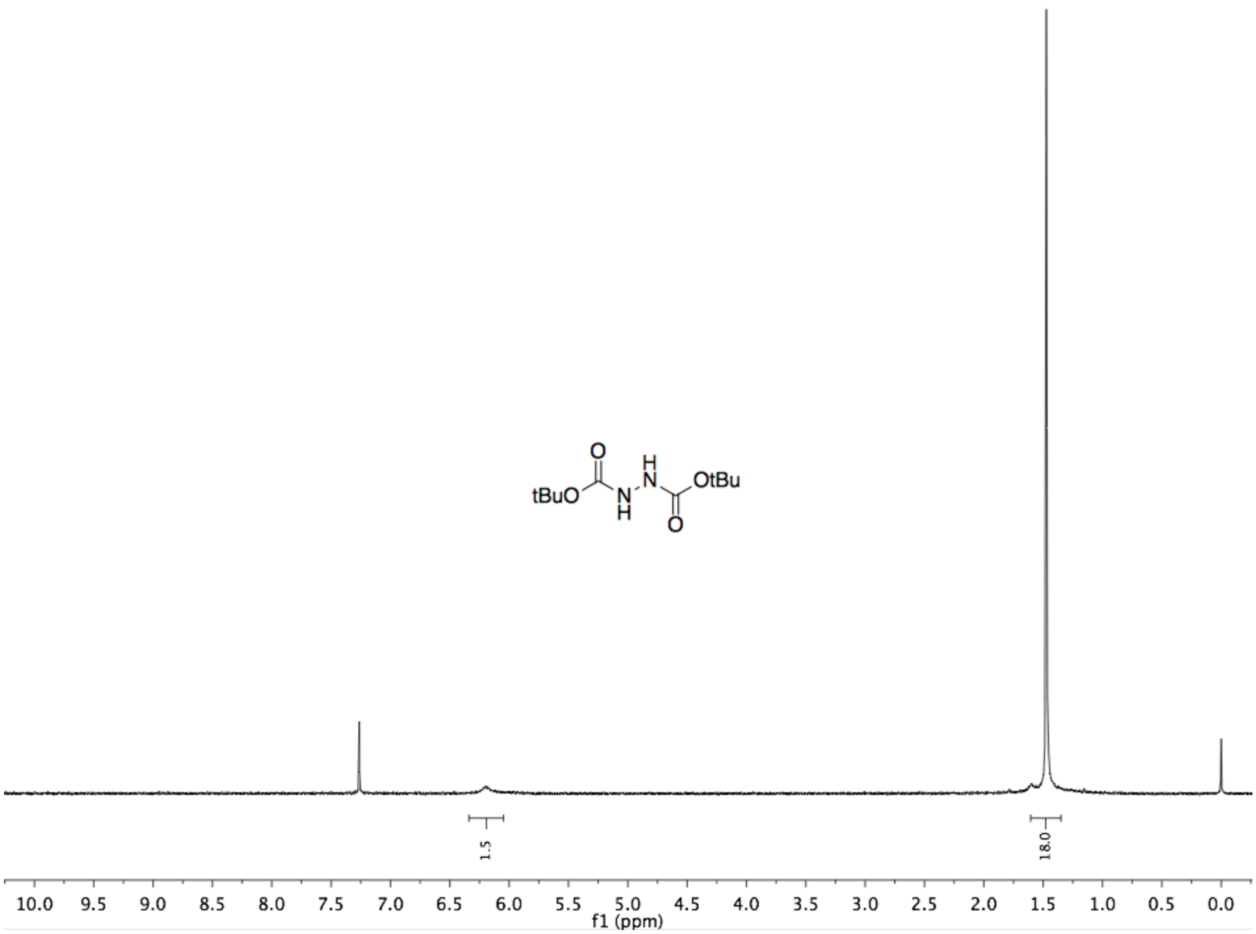

^{1}H NMR Spectrum of 11

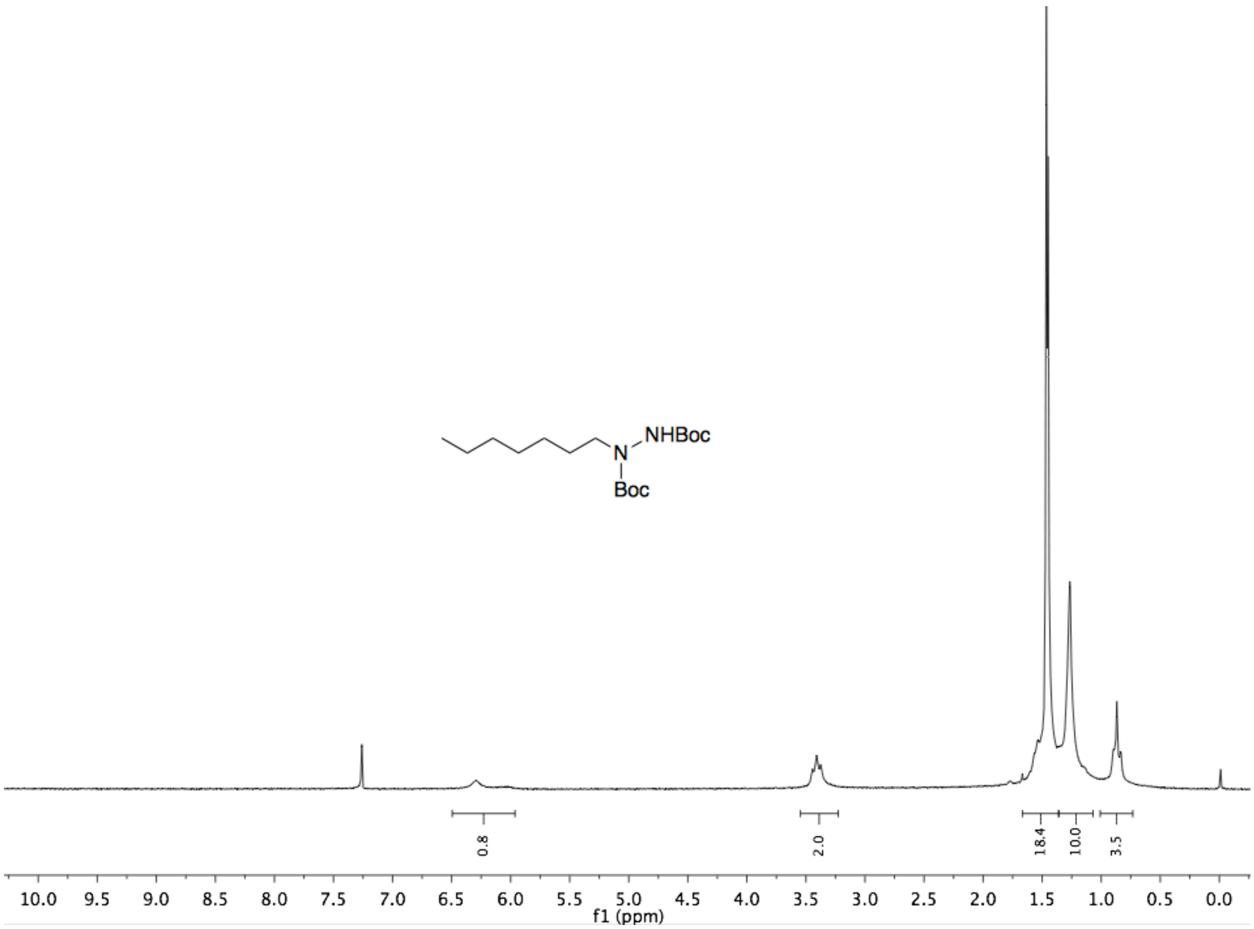

^{13}C NMR Spectrum of 11

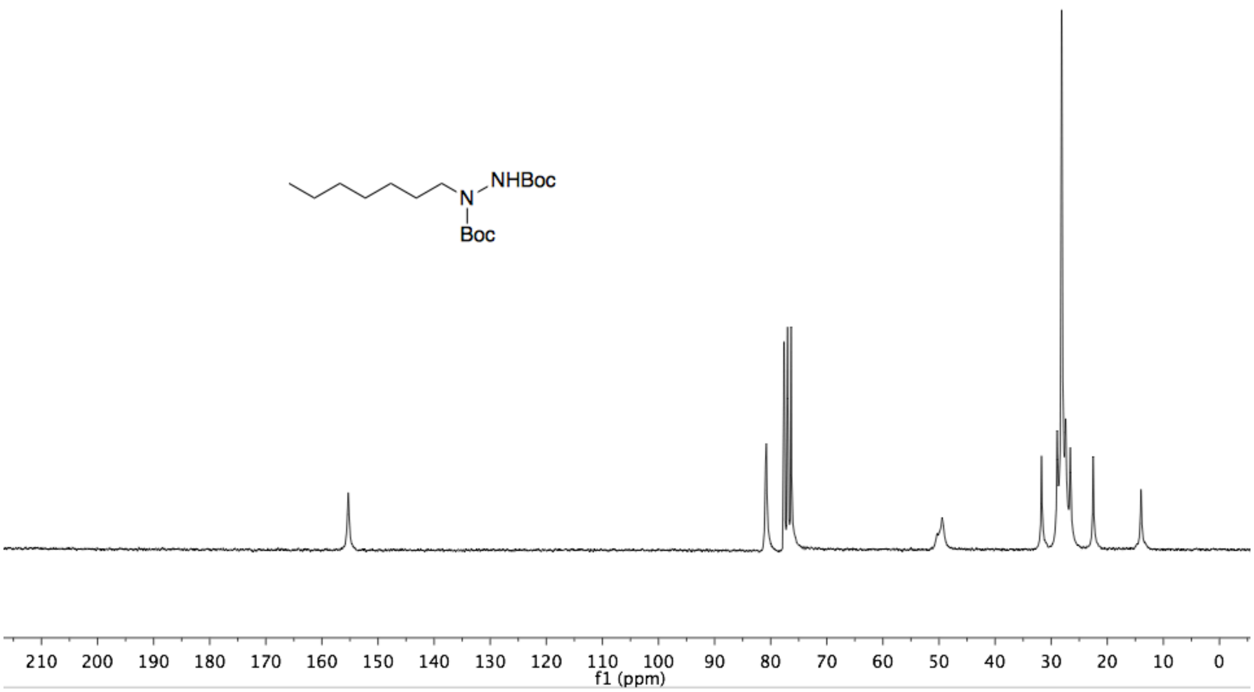

^{1}H NMR Spectrum of 14

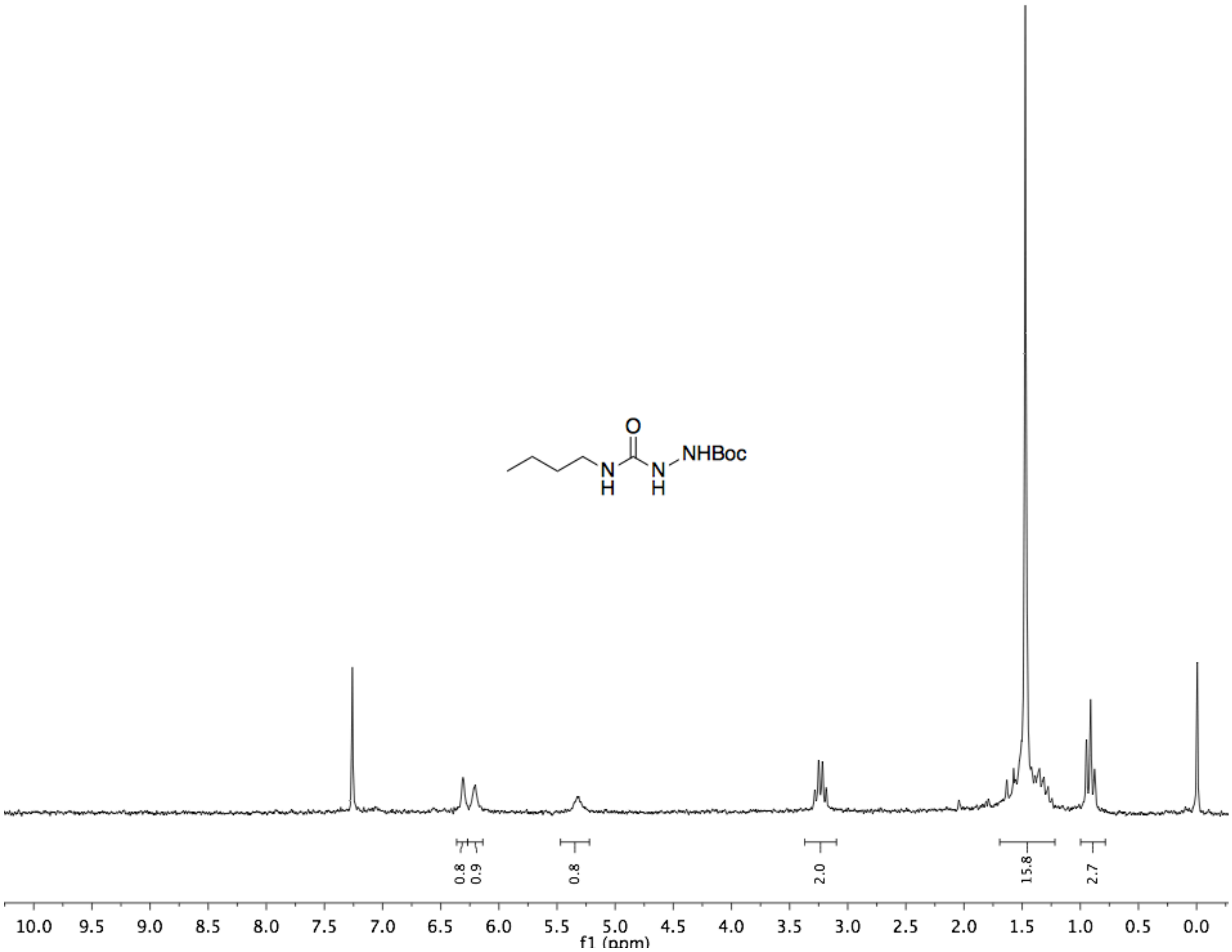

^{13}C NMR Spectrum of 14

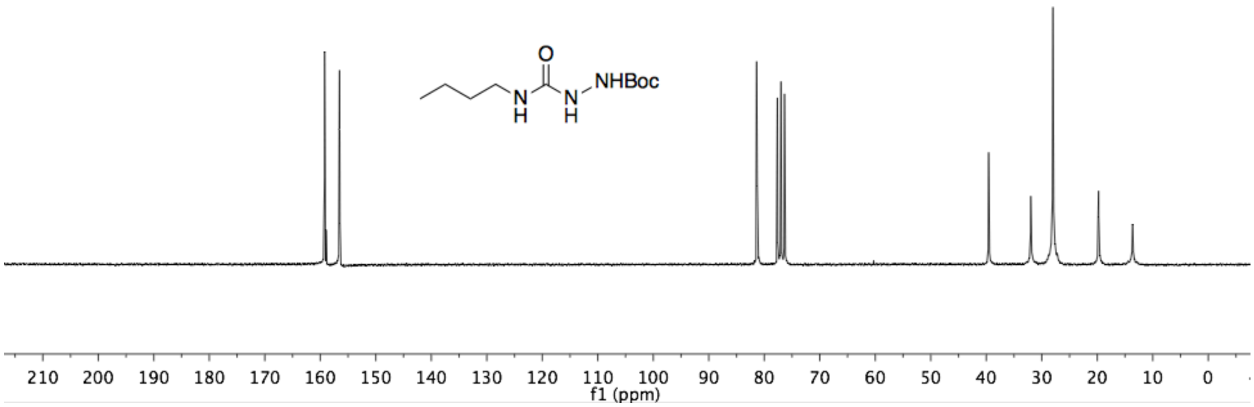

Supplement: IENZ_1265518_Supplementary_Material.pdf [file IENZ_A_1265518_SM2559.pdf]
